# Circulating retinol and 25(OH)D contents and their association with symptoms in children with chronic tic disorders

**DOI:** 10.1007/s00787-023-02226-4

**Published:** 2023-05-11

**Authors:** Cheng-Xin Wang, Bing Wang, Jian-Jian Sun, Chun-Ying Xiao, Huan Ma, Fei-Yong Jia, Hong-Hua Li

**Affiliations:** 1 Department of Developmental and Behavioral Pediatrics, The First Hospital of Jilin University, Changchun, 130021 Jilin China; 2 School of Public Health, Jilin University, Changchun, Jilin China; 3 Department of Laboratory Medicine, The First Hospital of Jilin University, Changchun, 130021 China

**Keywords:** Chronic tic disorders, Vitamin A, Vitamin D, Attention deficit hyperactivity disorder, Children

## Abstract

The present study measured serum levels of vitamin A (VA) and vitamin D (VD) in children with chronic tic disorders (CTD) and investigated their potential association with CTD and comorbidity of attention deficit hyperactivity disorder (ADHD) and the association of their co-insufficiencies or deficiencies with CTD symptoms. A total of 176 children (131 boys and 45 girls, median age of 9 years) with CTD were recruited as the CTD group. During the same period, 154 healthy children were selected as the healthy control (HC) cohort. Circulating retinol and 25-hydroxyvitamin D (25[OH]D) levels were measured for all participants using high-performance liquid chromatography (HPLC) and tandem mass spectrometry. The Yale Global Tic Severity Scale (YGTSS) was employed for the assessment of tic status and CTD impairment. The Swanson, Nolan, and Pelham Rating Scale (SNAP-IV) and the Children’s Yale-Brown Obsessive–Compulsive Scale (CY-BOCS) were used to evaluate comorbidity symptoms. CTD pediatric participants exhibited markedly diminished circulating retinol and 25(OH)D levels compared to HCs. Moreover, VA and VD deficiencies and their co-insufficiencies/deficiencies were more prevalent in CTD participants than HCs. Circulating 25(OH)D levels were inversely proportional to the YGTSS motor tic scores. YGTSS scores in CTD children with only VA or VD insufficiency or deficiency or with VA and VD co-insufficiency/deficiency did not differ from those in CTD children with normal VA and VD. CTD children with comorbid ADHD displayed reduced circulating retinol and 25(OH)D concentrations and elevated prevalence of VD deficiency compared to CTD participants without comorbid ADHD. Lower serum retinol content was intricately linked to the presence of elevated CTD and comorbid ADHD. VA and VD deficiencies and their co-insufficiencies/deficiencies were markedly enhanced in CTD pediatric participants compared to HCs. Lower VA concentration was linked to the presence of enhanced CTD and comorbid ADHD. Therefore, children with CTD, especially with comorbid ADHD, may be at a higher risk of VA or VD deficiency, which may prompt the clinicians to consider whether blood tests for VA and VD in CTD children would be helpful for clinical care.

**Supplementary Information:**

The online version contains supplementary material available at 10.1007/s00787-023-02226-4.

## Introduction

Chronic tic disorders (CTDs) are neurodevelopmental disorders that occur during the early years of human development, with motor and/or vocal tic symptoms for at least 1 year. CTD can be classified into three main categories: chronic motor tic disorder, chronic vocal tic disorder, and Tourette syndrome (TS) [1]. The first two categories do not necessarily include both motor and vocal tics. TS is the most drastic form of CTD, presenting as several motor tics with one or more vocal tics. In children and adolescents, the predicted incidence of CTD is 1–2%, and that of TS is 0.3–0.8%, with a male to female ratio of 3–4:1 [2, 3]. The majority of individuals with CTD (approximately 40–80%) have associated neuropsychiatric comorbidities, of which attention deficit hyperactivity disorder (ADHD) and obsessive–compulsive disorder (OCD) are the most prevalent [4, 5]. Persistent tic symptoms and their comorbidities, especially ADHD, seriously influence the quality of life and socioeconomic ranking of CTD individuals [6–8].

The exact etiology of CTD has not yet been fully clarified. Recent studies have suggested that CTD is caused by both polygenetic and environmental agents [9–11]. The heritability of CTD is between 0.25 and 0.77 [12, 13]. Susceptibility genes have been associated with several neurotransmitter and neuroimmune systems. Environmental factors, such as dietary composition, potentially pathogen infection, psychosocial stress, exercise, and sleep, may interact with susceptibility genes to increase CTD susceptibility and influence the severity of tic symptoms [14–18].

One environmental factor of interest is micronutrients, such as fat-soluble vitamins A and D. Vitamin D (VD) is a neurosteroid hormone that is mainly synthesized by subcutaneous 7-dehydrocholesterol under sunlight or from vitamin D2 in some foods. In addition to maintaining skeletal health, it plays an important role in neurodevelopment and immune function [19]. VD can regulate the expression of more than 200 genes [20]. Vitamin D receptor (VDR) and its activating enzyme 1α -hydroxylase exist widely in the human brain, and it is speculated to possess neurotrophic and neuroprotective properties, affecting neuroplasticity, neurotransmission, and neuroimmunity [19, 21].

Striatal dopaminergic dysregulation is regarded as a commonly discussed possible mechanism of CTD pathophysiology [22]. Pathophysiological study showed that the impaired cortico-basal ganglia network, whereby dopaminergic input from the substantia nigra pars compacta regulates function across the basal ganglia, is strongly correlated with CTD pathogenesis [23]. VD protects the dopamine system via gene expression regulation [24, 25], and it also modulates relevant enzymes: tyrosine hydroxylase and catechol aminotransferase, which are the rate-limiting enzymes for dopamine, norepinephrine, and adrenaline syntheses [26]. Hence, VD deficiency can result in dopaminergic dysregulation within CTD participants. Additionally, VD is closely related to brain development [19], and abnormal brain structure and maturation are also considered parts of the pathogenesis of CTD [27]. Therefore, VD deficiency might be related to the pathogenesis of CTD.

Vitamin A (VA) is also a crucial micronutrient, and its active form, retinoic acid (RA), is intricately involved in central nervous system-based cellular development and differentiation. Animal investigations have revealed that VA deficiency impairs synaptic plasticity in the hippocampus, which is modulated by retinoic acid nuclear receptor-α [28]. Moreover, VA and VD synergistically regulate gene expression [29]. VD and retinoic acid interact with VDR and retinoic acid receptor (RXR) complex in the nucleus for further gene regulation [30]. Therefore, VA deficiency might contribute to CTD pathogenesis.

Our previous studies demonstrated an association between VD deficiency and CTD [31, 32]. Recently, a large case–control study (n = 2960) in China reported reduced concentrations of 25-hydroxyvitamin D (25[OH]D) in children with tics versus healthy controls, but no association was observed between these levels and the degree of tic [33]. A multicenter study revealed no obvious differences between VD concentrations and an elevated prevalence or degree of tics; however, the study found that VD levels were intricately linked to the prevalence and degree of comorbid ADHD in children and adolescents with CTD [34]. Considering the mechanistic synergy of VA and VD and their possible association with neuropsychiatric disorders, our previous study also found a correlation between VA and VD levels with ADHD [35], although it did not explore the relationship between VA and CTD. To date, only one study (n = 59) has reported the relationship between VA levels and tics and found that VA deficiency was associated with tics in children [36].

Based on the inconsistencies of the above-mentioned results, those findings require further study. Here, we assessed VA and VD levels in CTD children aged 5–12 years and delineated their correlations with CTD symptoms and ADHD comorbidity.

## Methods

### Participants and procedures

From May 2017 to June 2020, we selected 176 CTD children aged 5–12 years who were evaluated for the first time in the Outpatient Department of the First Hospital of Jilin University. Initially, all children suspected of having CTD were examined by reviewing their current health, developmental and family history, along with a thorough physical evaluation and parental interviews by a developmental pediatrician referencing the CTD criteria based on the Diagnostic and Statistical Manual of Mental Disorders (DSM-5) [1]. A total of 176 children fulfilled the diagnostic criteria for CTD, including 131 boys and 45 girls, with a median age of 9 years (range: 5–12 years). During the same period, 154 healthy children without any somatic and behavioral abnormalities were recruited into the healthy control (HC) group from the same outpatient department, including 109 boys and 45 girls, with a median age of 9 years (range: 6–12 years). These participants did not present any signs of neurodevelopmental or other disorders that may influence micronutrient homeostasis.

Exclusion criteria comprised (1) children suffering from other neurological or neurodevelopmental disorders, such as neurodegenerative disorders, autism, epilepsy, and intellectual disorders; (2) children with severe picky eating, malnutrition, digestive diseases, etc., that may affect micronutrient absorption and metabolism; (3) children who had received VA, VD, or multivitamin supplements in the past 3 months; (4) abnormal liver and kidney function, or taking glucocorticoids, benzodiazepines, and other medication that may influence VA and VD metabolism. This study used a case–control design. The parents or guardians of the children signed documented informed consent before the initiation of the study. This work received ethical approval and registry from the First Hospital of Jilin University and the Chinese Clinical Trial Registry (registration number: CHICTR-OPC-17013502), respectively. The authors of the present study previously published results involving 46 cases, with 42 healthy controls enrolled in this study [31].

### Initial clinical assessment

The parents or caregivers of all participants completed a survey developed by our research team regarding demographic characteristics, including sex, age, place of residence, sun exposure, physical activity, daily diets, and vitamin supplementation. An electronic scale and stadiometer were used to measure the children’s height and weight. Body mass index (BMI) was computed as follows: $\mathrm{BMI }=\mathrm{ weight }(\mathrm{kg})/(\mathrm{height }(\mathrm{m})2$. Based on the age percentile of BMI, we divided the participants into three categories: normal weight (< 85th percentile), overweight (85–95th percentile), or obese (> 95th percentile) [37].

### Behavioral assessments

The Yale Global Tic Severity Scale (YGTSS) was employed for tic degree assessment and impairment in children in the CTD group. The YGTSS is a semi-structured scale assigning scores based on frequency, intensity, quantity, interference, and complexity of tics [38]. The sum tic score includes motor and vocal tic scores, with a range from 0 to 50 points. The YGTSS impairment ranking subscale is designed to assess functional impairments related to tics, such as psychological and self-esteem impairment, with scores between 0 and 50 points. The total score of YGTSS is the sum of the total tic scores and the impairment rating subscale scores. The Chinese version of YGTSS with good psychometric properties was utilized in this study [39]. Additionally, the parent and teacher versions of the Swanson, Nolan, and Pelham Rating Scale (SNAP-IV) were employed to evaluate the presence of comorbid ADHD [40]. The Children’s Yale–Brown Obsessive–Compulsive Scale (CY-BOCS) evaluated the OCD symptoms [41]. A self-made behavior questionnaire was used to screen for anxiety, depression, and other emotional problems. Those who scored positive (meeting the symptom criteria for ADHD or OCD) on the SNAP-IV or CY-BOCS would be confirmed by the developmental pediatrician according to the criteria for ADHD and OCD in the DSM-5 to make the final diagnosis of whether the patient is comorbid with ADHD or OCD.

### Laboratory measurements

After enrollment, CTD participants underwent a routine blood test, which included blood cell count, rheumatoid factor, anti-O antibody, liver and kidney function, serum calcium level, and zinc, iron, copper, and magnesium levels. Serum retinol and 25(OH)D content of both groups were measured at baseline by high-performance liquid chromatography and tandem mass spectrometry (HPLC-MS/MS) in Jilin Hehe Medical Center (Clinical Laboratory). Circulating retinol levels of > 1.05, 0.7–1.05, and < 0.7 μmol/L were judged as “normal VA”, “marginal VA”, and “VA deficiency”, respectively [42]. Serum 25(OH)D concentrations of 30–90, 21–29, and < 20 ng/mL were judged as “optimal VD”, “VD insufficiency” and “VD deficiency” respectively [43]. In the present study, we have defined the VA deficiency by a retinol level < 0.7 μmol/L and VA insufficiency 0.7–1.05 μmol/L. Similarly, we defined the VD deficiency by a 25(OH)D level < 20 ng/mL and VD insufficiency 21–29 ng/mL.

In the United States, Boston (MA) has the same latitude as Changchun, which is between 42° and 45° of North latitude. Thus, Summer (June–October) and winter seasons (November–May) were used for the division of the year in the present study, adopting the results presented by the Boston study [44] regarding the association between the seasonal variation of sunlight and serum 25(OH)D content.

### Statistical analysis

All data were analysed by SPSS V23.0 statistical software. The Kolmogorov–Smirnov test was employed for the normality assessment of continuous variables. Continuous information was represented by P50 (P25, P75). Categorical data were expressed as frequencies with percentages. Using the nonparametric Mann–Whitney U test, the continuous or ordered categorical variables of both groups were compared. A chi-squared test was utilized to assess differences in the amount of unordered categorical variables of both groups. The Kruskal–Wallis H test was used to compare the differences in the total and subscale scores of YGTSS among the four groups, and the Mann–Whitney U test was further used to conduct a post-hoc test between the two groups. Spearman’s correlation coefficient was employed to examine the correlation between YGTSS total scores and subscale scores and serum retinol and 25(OH)D concentrations. Multivariate logistic regression analysis was employed to determine the association between circulating retinol and 25(OH)D concentrations and the presence of CTD and ADHD comorbidity. The odds ratio (OR) was reported in the result. The significance threshold was set to *P* of < 0.05, with two-sided tests for all analyses.

## Results

### Comparing the sociodemographic characteristics between CTD and HC participants

The present study enrolled 176 children with CTD. Eighty-one participants presented with a chronic motor tic disorder, 14 participants presented with a chronic vocal tic disorder, and 81 participants presented with TS. Seventy children were comorbid with ADHD, six children were comorbid with OCD, and 13 had anxiety symptoms.

Table 1 provides a detailed breakdown of sociodemographic data for these two groups. No discernible differences were observed between CTD and HC children in sex, age, BMI, parents’ education level, resident area, the season of blood collection, annual household income, and daily exposure to sunlight. Compared with HCs, the proportion of CTD children with a dairy intake of > 300 mL was significantly reduced.

**Table 1:** Comparison of sociodemographic characteristics between the CTD and HC group^a^

Variable	CTD (n = 176)	Control (n = 154)	*χ*^*2*^*/Z*	*P*
Sex			0.55	0.46
Boys	131 (74.4%)	109 (70.8%)		
Girls	45 (25.6%)	45 (29.2%)		
Age (years)	9 (8, 10)	9 (8, 10)	− 1.69	0.09
BMI			0.47	0.79
< 85th percentile	135 (76.7%)	114 (74.0%)		
85–95th percentile	26 (14.8%)	27 (17.5%)		
> 95th percentile	15 (8.5%)	13 (8.4%)		
Father’s level of education			0.28	0.60
High school or below	92 (52.3%)	76 (49.4%)		
Bachelor’s degree and above	84 (47.7%)	78 (50.6%)		
Mother’s level of education			0.78	0.38
High school or below	67 (38.1%)	66 (42.9%)		
Bachelor’s degree and above	109 (61.9%)	88 (57.1%)		
Residence			0.23	0.63
Urban area	125 (71.0%)	113 (73.4%)		
Rural area	51 (29.0%)	41(26.6%)		
Season of blood collection			0.005	0.94
Winter	93 (52.8%)	82 (53.2%)		
Summer	83 (47.2%)	72 (46.8%)		
Annual household income (¥)			0.34	0.85
< 100,000	11 (6.3%)	10 (6.5%)		
100,000–250,000	137 (77.8%)	123 (79.9%)		
> 250, 000	28 (15.9%)	21 (13.6%)		
Daily intake of dairy products (mL)			9.91	0.002
0–300	147 (83.5%)	106 (68.8%)		
> 300	29 (16.5%)*	48 (31.2%)		
Daily exposure to sunlight (h)			1.17	0.56
≤ 1 h	71 (40.3%)	71 (46.1%)		
1–2 h	80 (45.5%)	62 (40.3%)		
> 2 h	25 (14.2%)	21(13.6%)		

*P* < 0.05 compared with the HC group

*CTD* chronic tic disorders, *BMI* body mass index

^a^n (%), P50(P25, P75)

### Serum retinol and 25(OH)D levels in the CTD and HC participants

Table 2 exhibits the status of serum retinol and 25(OH)D in both groups. The median circulating concentration of retinol and 25(OH)D in CTD participants was markedly diminished compared to HCs. CTD participants were more likely than healthy control participants to be retinol marginal or deficient. Compared to HC participants, remarkably more CTD participants had 25(OH)D deficiency and serum retinol and 25(OH)D co-insufficiency/deficiency.

**Table 2:** Comparison of serum retinol and 25(OH)D levels between the CTD and HC group^a^

Status	CTD (n = 176)	Control (n = 154)	*Z/χ*^*2*^	*P*
Serum retinol, (umol/L)	1.09 (1.02,1.23)*	1.23 (1.05,1.40)	− 4.13	0.001
Normal	94 (53.4%)	111 (72.1%)	− 3.59	< 0.001
Marginal	78 (44.3%)*	43 (27.9%)		
Deficiency	4 (2.3%)*	0 (0.0%)		
Serum 25(OH)D, (ng/mL)	21.7 (16.6, 27.8)*	24.1 (19.4, 28.8)	− 2.63	0.01
Optimal	31 (17.6%)	32 (20.8%)	− 2.27	0.02
Insufficient	70 (39.8%)	78 (50.6%)		
Deficiency	75 (42.6%)*	44 (28.6%)		
VA and VD co-insufficiency/deficiency^b^	69 (39.2%)*	38 (24.6%)	7.91	0.005

*CTD* chronic tic disorders

^a^n (%), P50(P25, P75)

^b^Serum retinol marginal or deficient and serum 25(OH)D insufficient or deficient

*P < 0.05 compared with the HC group

### Association between circulating VA and VD levels with YGTSS scores

The association coefficients of circulating retinol and 25(OH)D with the sum YGTSS and subscale scores are provided in Table 3. The circulating 25(OH)D level was significantly and negatively associated with the motor score. No obvious correlation was observed between serum retinol level and YGTSS total score and subscale score.

**Table 3:** Correlation between serum retinol and 25(OH)D concentrations with CTD symptoms

YGTSS score	Serum retinol (umol/L)	Serum 25(OH)D (ng/mL)
YGTSS total score	− 0.08	− 0.14
Total tic score	− 0.14	− 0.11
Motor score	− 0.06	− 0.24^*^
Vocal score	− 0.12	0.10
Impairment	0.14	− 0.06

*YGTSS* The Yale Global Tic Severity Scale

*P < 0.05

### Relationships between VA and VD co-insufficiency/deficiency and YGTSS scores

Among CTD participants, 69 (39%) exhibited VA and VD co-insufficiency/deficiency, 13 cases (7%) exhibited VA insufficiency or deficiency only, 76 cases (43%) exhibited VD insufficiency or deficiency only, and 18 cases (10%) exhibited normal VA and VD.

Marked differences were observed in the total tic and motor scores in YGTSS between the VA and VD co-insufficiency/deficiency, VA insufficiency or deficiency only, VD insufficiency or deficiency only, and VA and VD normal participants. The total tic score of VA and VD co-insufficiency/deficiency participants were markedly elevated compared to the VA insufficiency or deficiency only (*Z* = − 2.28, *P* = 0.02) and VD insufficiency or deficiency only participants (*Z* = − 3.04, *P* = 0.002). The motor score of VA and VD co-insufficiency/deficiency participants were markedly elevated compared to the VA insufficiency or deficiency only participants (*Z* = − 2.44, *P* = 0.015). However, there was no significant difference in the total tic and motor scores between the VA and VD co-insufficiency/deficiency group and the VA and VD normal group. Although the total tic and vocal scores in the VA insufficiency or deficiency only and VD insufficiency or deficiency only participants were lower than those in the VA and VD normal participants, these did not reach significance (Table 4).

**Table 4:** Associations between VA and VD co-insufficiency/deficiency and YGTSS scores^a^

Scores	VA and VD co-insufficiency/deficiency (n = 69)	VA insufficiency or deficiency only (n = 13)	VD insufficiency or deficiency only (n = 76)	VA and VD normal (n = 18)	*H*	*P*
YGTSS total scores	30 (24, 35)	22 (20, 30)	26 (22, 33)	30 (21, 42)	6.40	0.09
Total tic score*	19 (14, 24)^#∮^	12 (10, 20)	15 (12, 20)	17 (11, 26)	11.38	0.01
Motor score*	13 (10,17)^#^	10 (3,13)	12 (10, 15)	12 (8, 15)	8.63	0.04
Vocal score	6 (0, 10)	5 (0,13)	0 (0, 8)	8 (0,12)	7.63	0.05

^a^P50(P25, P75)

*Significantly different among the four groups, *P* < 0.05

^#^*P* < 0.05 compared with the VA insufficiency or deficiency only group

^∮^*P* < 0.05 compared with the VD insufficiency or deficiency only group

### Serum retinol and 25(OH)D levels in CTD-comorbid ADHD and CTD without comorbidity participants

In the CTD group, 70 children had ADHD comorbidity, 19 cases had anxiety and obsessive–compulsive symptoms, and the remaining 87 cases had no comorbidities.

Table 5 shows the serum VA and VD contents in CTD participants without comorbidity and children with comorbid ADHD. Median serum concentrations of retinol and 25(OH)D in the co-ADHD group were markedly diminished compared to CTD participants without comorbidity. The proportion of children with 25(OH)D deficiency in co-ADHD participants was markedly increased compared to non-comorbidity participants, and the proportion of children with optimal 25(OH)D in co-ADHD participants was drastically reduced compared to non-comorbidity participants. No obvious difference was seen in the proportion of circulating retinol normal and marginal levels and deficiency between the two groups.

**Table 5:** Comparison of serum retinol and 25(OH)D levels between the CTD without comorbidity group and CTD comorbid ADHD group^a^

Status	CTD without comorbidity (n = 87)	CTD comorbid ADHD (n = 70)	*Z*	*P*
Serum retinol, (umol/L)	1.09 (1.02, 1.26)	1.03 (0.98, 1.16)*	− 2.45	0.015
Normal	47 (54.1%)	30 (42.9%)	− 1.37	0.17
Marginal	38 (43.6%)	38 (54.3%)		
Deficiency	2 (2.3%)	2 (2.8%)		
Serum 25(OH)D, (ng/mL)	23.8 (17.5, 30.4)	18.9 (15.3, 24.2)*	− 3.15	0.002
Optimal	22 (25.3%)	4 (5.7%)*	− 3.85	< 0.001
Insufficient	37 (42.5%)	25 (35.7%)		
Deficiency	28 (32.2%)	41 (58.6%)*		

*CTD* chronic tic disorders, *ADHD* attention deficit hyperactivity disorder

^a^n (%), P50(P25, P75)

*P < 0.05 compared with the CTD without comorbidity group

### Correlations between circulating VA and VD contents and CTD presence

Based on the multivariate analysis, circulating retinol concentration (adjusted OR 0.19; 95% confidence interval, 0.07–0.53; *P* = 0.002) and daily dairy intake (> 300 mL) (0.46; 0.27–0.79; 0.004) were significantly negatively correlated with the presence of CTD after adjusting for BMI and sunshine exposure (Table 6).

**Table 6:** The correlation between serum retinol and 25(OH)D levels and the presence of CTD

Variable	*β*	Adjusted OR (95% CI)	*P*
Serum retinol, (umol/L)	− 1.64	0.19 (0.07–0.53)	0.002
Serum 25(OH)D, (ng/mL)	− 0.02	0.98 (0.96–1.01)	0.21
BMI			
< 85th percentile			
85–95th percentile	− 0.08	0.92 (0.49–1.72)	0.80
> 95th percentile	0.07	1.07 (0.47–2.46)	0.87
Daily intake of dairy products, (mL)			
0–300			
> 300	− 0.78	0.46 (0.27–0.79)	0.004
Daily exposure to sunlight, (h)			
0–1			
1–2	0.28	1.32 (0.81–2.15)	0.27
> 2	0.26	1.30 (0.65–2.60)	0.46

*OR* odds ratio; outcome variable, CTD presence (1 = yes, 0 = no) (*P* < 0.05)

### Correlations between circulating VA and VD contents and ADHD comorbidity presence

Based on the multivariate analysis, circulating retinol content (adjusted OR 0.11; 95% confidence interval, 0.02–0.71; *P* = 0.02) was significantly negatively correlated with the presence of ADHD comorbidity, and age (3.31; 2.09–5.25; < 0.001) was significantly positively correlated with the presence of ADHD comorbidity after adjusting for BMI, daily dairy product intake, and daily sunlight exposure (Table 7).

**Table 7:** The correlation between serum retinol and 25(OH)D levels and the presence of ADHD comorbidity

Variable	*β*	Adjusted OR (95% CI)	*P*
Serum retinol, (umol/L)	− 2.25	0.11 (0.02–0.71)	0.02
Serum 25(OH)D, (ng/mL)	− 0.04	0.96 (0.92–1.01)	0.16
Age. (years)	1.20	3.31 (2.09–5.25)	< 0.001
BMI			
< 85th percentile			
85–95th percentile	− 0.65	0.52 (0.15–1.88)	0.32
> 95th percentile	− 0.22	0.80 (0.17–3.80)	0.78
Daily intake of dairy products, (mL)			
0–300			
> 300	0.06	1.07 (0.33—3.46)	0.92
Daily exposure to sunlight, (h)			
0–1			
1–2	0.54	1.72 (0.72–4.11)	0.23
> 2	− 0.47	0.62 (0.18–2.12)	0.45

*OR* odds ratio; outcome variable, presence of ADHD comorbidity. (1 = yes, 0 = no) (*P* < 0.05)

## Discussion

This research presents four major findings. First, serum VA and VD contents in CTD participants were drastically reduced compared to HCs, and VA and VD deficiency and their co-insufficiency/deficiency prevalence was markedly elevated compared to HCs. Second, YGTSS scores in CTD children with only VA or VD insufficiency/deficiency or with VA and VD co-insufficiency/deficiency did not differ from those in CTD children with normal VA and VD. Third, CTD participants with comorbid ADHD had lower serum VA and VD contents and higher VD deficiency prevalence compared with CTD participants without comorbid ADHD. Fourth, lower serum VA contents were correlated with the presence of enhanced CTD and comorbid ADHD.

Our results found lower levels of VD and higher prevalence of VD deficiency in children with CTD, and the serum VD levels were negatively correlated with tic symptoms. These findings are consistent with our previous research [31, 32]. Recently, a large sample case–control study in China has also shown that serum VD levels of participants with tic disorders were drastically reduced compared to HC, and VD deficiency prevalence was markedly increased. However, no correlation was found between serum VD levels and tic symptoms [33]. The possible reasons for the inconsistent results compared to the current study are as follows: differences in sample size, geographical location of the population, and the classification of tic disorders. A key limitation of Wang et al. [33], as noted by the authors themselves, was that they did not analyze the possible effects of different types of tic disorders and comorbidities on VD levels. In contrast to our results, Bond et al. found that higher serum VD levels increased the risk of CTD presence and did not correlate with tic severity [34]. The present study found a negative correlation between serum VD levels and motor tic scores. These different findings may be related to ethnicity and genetic susceptibility. Another possible reason for the difference is that the control group of Bond et al. was a group of individuals who did not develop tics during up to 7 years of follow-up [34]. Diet, vitamin D supplementation, and outdoor activities during follow-up may influence the final outcome-tic onset. One limitation of the study of Bond et al. as mentioned by the authors themselves, was the lack of data on vitamin D supplementation and sun exposure [34]. In addition, the age range of the study population was 5–12 years old in the present study and 3–16 years old in the study conducted by Bond et al. [34]. Different nutrition, diets, and outdoor exercise at different ages may affect VD status. This suggests that future global multicenter studies should be conducted to elucidate the relationship between VD and CTD.

The mechanism of lower VD levels in children with CTD is unclear. VD is a neurosteroid hormone produced mainly by ultraviolet B (UVB) radiation to the skin and is also naturally found in some foods. Its formation was found to be modulated by season, latitude, air pollution, skin pigment, diet, and certain drugs that may affect the absorption or metabolism of VD [21]. The study also found that the daily intake of dairy products in children with CTD was significantly lower than that of HCs, which might indicate that CTD children might have abnormal diet structure. For example, children who are picky eaters may not like drinking dairy products, resulting in an insufficient intake of VD food sources (as dairy products in China usually contain VD). Therefore, decreased dairy intake is a possible influencing factor for lower VD levels in children with CTD. However, since the present study does not conduct a detailed investigation of dietary behavior problems among the participants, the relationship between dairy intake and VD levels needs to be further studied.

Although the current study could not establish a causal relationship between lower VD levels and CTD pathogenesis, it did suggest that VD was associated with CTD to some extent. VD is essential for the normal development of dopaminergic neurons. Cortico-basal ganglia circuit dopamine dysfunction is considered as a commonly discussed possible mechanism of CTD pathophysiology [22]. In vitro and in vivo studies have found that VD could regulate the genetic profile of dopamine rate-limiting enzyme–tyrosine hydroxylase [26, 45]. Moreover, VD modulates the levels of glial cell line neurotrophic factor (GDNF) and GDNF receptor C-Ret, which are essential for the survival and differentiation of dopaminergic neurons [46]. Therefore, as one of the environmental factors, VD deficiency might increase the genetic susceptibility to CTD during the critical period of brain development, thereby increasing the risk of CTD onset. Neuroinflammation caused by the immune mechanism is also believed to be involved in the pathophysiological process of CTD [47]. VD plays an anti-inflammatory role by regulating cellular and humoral immune responses, and levels of VD have been associated with elevated inflammatory markers in CTD participants [21, 47]. VD deficiency potentially affect the severity of neuroinflammation. A small sample of VD supplementation (n = 36) in our previous study has also suggested that VD supplementation was effective in improving tic symptoms and further indicated that lower levels of VD might contribute to CTD pathogenesis [31].

In this study, CTD participants had lower circulating retinol concentrations and a higher prevalence of VA insufficiency or deficiency (approximately 46%) compared to HCs. To date, only one case–control investigation [36] has reported the correlation between circulating VA concentration and TD in children. The study’s results showed that serum VA content in TD participants was markedly reduced compared to HCs, and lower VA level was associated with increased tic symptoms [36]. In contrast, our study found no association between circulating VA levels and symptoms in CTD participants. This difference might be related to different populations that were included. There were 59 cases in the observation group of the above-mentioned study, among which only 34 cases were CTD children.

RA, an active derivative of VA, is involved in cell differentiation, antioxidant activity, inflammation, and neuronal plasticity and critically modulates normal activities of the central nervous system. Many studies have demonstrated striatal dopaminergic dysfunction and striatal microstructural abnormalities in CTD participants [22, 48]. RA can affect the dopamine signaling pathway and its plasticity by regulating gene transcription [49, 50]. RA receptors are widely available in striatal neurons [51]. RA signaling also modulates neurogenesis and striatal neuron differentiation [52–54]. Animal investigations have shown that VA supplementation improved voluntary movements in 6-hydroxydopamine (6-OHDA) lesioned rats by increasing D2 and RXR receptor expressions within the striatum [55]. RA therapy alleviates L-DOPA-induced dyskinesia movements in mice by upregulating striatal MOR1 levels and signaling [52]. These symptoms are partly similar to tics. In addition, RA and its mediated signaling pathway are related to intestinal inflammation [56], and the changes in the RA pathway may be related to intestinal immune inflammation and microbial homeostasis imbalance [57, 58]. Particular gut microbiota changes could be observed in children with TS [59]. Fecal transplantation can alleviate tic severity in a TS mouse model [60], and the RA signaling pathway might play a mediating role in this process. Therefore, we speculate that VA may be involved in the pathophysiological mechanism of CTD.

The present study found that lower serum VD levels were associated with higher motor tic scores. Considering the synergistic effect of VA and VD in the nucleus (RA and VD must interact with the RXR and VDR complex to exert gene regulation) [30, 61], we explored the relationship between VA and VD co-insufficiency/deficiency and tic symptoms. However, contrary to our hypothesis, the subgroup analysis showed no difference in YGTSS scores among CTD children with only VA or VD insufficiency/deficiency or with VA and VD co-insufficiency/deficiency compared to CTD children with normal VA and VD. Conversely, CTD children with only VA or VD insufficiency/deficiency appear to have lower total tic and vocal tic scores than CTD children with normal VA and VD, although these did not reach significance. In summary, the present results suggest that VA or VD only insufficiency/deficiency or VA and VD co-insufficiency/deficiency are not associated with tic symptoms. Tic symptoms and their fluctuation are affected by sex, age, dietary factors, and exercise. However, due to the limitation of the sample size, we failed to match the four groups with sex, age, daily dairy intake, and daily exposure to sunlight. Therefore, future studies should expand the sample size and control for these factors to further explore the relationship between VA and VD co-insufficiency/deficiency and tic symptoms.

For comorbid ADHD in children with CTD, we found lower serum VA and VD levels and a higher prevalence of VD deficiency compared with that of non-ADHD comorbidity. ADHD is a common comorbidity of CTD, and the prevalence of comorbid ADHD in children with CTD is 40–80% [62]. Two recent meta-analyses, as well as one of our previous studies, have also found a reduced concentration of 25(OH)D in ADHD participants compared to HCs [35, 63, 64]. However, limited investigations have evaluated the role of 25(OH)D within the context of comorbid ADHD in CTD participants. Thus far, only one study has explored the relationship between VD and ADHD comorbidity in children with CTD and found that lower VD levels were linked to the presence and degree of comorbid ADHD in CTD participants [34]. Although our study found that the CTD comorbid with ADHD group had lower VD levels and a higher prevalence of VD deficiency, we observed no discernible association between lower VD contents and the presence of comorbid with ADHD in CTD children, which may be related to racial differences and dietary structure, needing to be further explored.

We assessed, for the first time, the relationship between VA and comorbidity of ADHD in CTD children and found that lower VA contents were correlated with the presence of CTD and ADHD comorbidity. Evidence of the genetic, neurophysiological, and neurobiological overlap between CTD and ADHD suggests that the two disorders are intricately intercorrelated [62]. Children with both CTD and ADHD have a greater functional impairment and lower quality of life [65]. Overall, we found that children with CTD had a higher risk of VA and VD deficiencies in our sample, which may prompt the clinicians to consider whether blood tests for VA and VD in children with tics would be helpful.

Although our investigation was based on a case–control design, and we matched the sex, age, blood collection time, BMI, and socioeconomic status of the two groups to circumvent the impact of confounding factors on VA and VD contents, there were still some limitations. First, VA is obtained mainly from food. Therefore, dietary behavior has a larger influence on participants’ VA content, and children with CTD may be more picky or partial eaters, thus, affecting the circulating VA level. However, we did not conduct a detailed investigation of eating behavior problems in the participants, nor did we analyze the possible influence of these factors on vitamin levels. Second, the daily intake of dairy products was not matched between the two groups, and the number of CTD children was significantly lower than HCs, which may also have led to biased results. The third limitation is that our cross-sectional study could only conclude that VA and VD levels were correlated with CTD and its comorbidities with ADHD—it could not infer a causal relationship between VA or VD deficiency and the presence of CTD and its comorbidities with ADHD. Prospective longitudinal investigations are warranted to establish whether reduced VA and VD contents predispose CTD to persistence or comorbidity of ADHD.

## Conclusion

VA and VD deficiencies and their co-insufficiencies/deficiencies in CTD participants were markedly increased compared to HCs. Lower VA concentrations were linked to the increased presence of CTD and comorbid ADHD. Thus, CTD children, particularly those with comorbid ADHD, may be at a higher risk of VA or VD deficiency, which may prompt the clinicians to consider whether blood tests for VA and VD in CTD children would be helpful for clinical care. However, VA and VD mechanisms in CTD require further investigation.

### Supplementary Information

Below is the link to the electronic supplementary material.

Supplementary file1 (XLS 116 KB)

Supplementary file2 (XLS 114 KB)

## Data Availability

The datasets used and analysed during the current study are included in the supplementary material, further inquiries can be directed to the corresponding author.

## Abbreviations

VA: Vitamin A
VD: Vitamin D
CTD: Chronic Tic Disorders
ADHD: Attention Deficit Hyperactivity Disorder
HC: Healthy Control
25[OH]D: 25-Hydroxyvitamin D
HPLC: High-performance Liquid Chromatography
YGTSS: Yale Global Tic Severity Scale
SNAP-IV: Swanson, Nolan, and Pelham Rating Scale
CY-BOCS: Children’s Yale-Brown Obsessive–Compulsive Scale
TS: Tourette Syndrome
OCD: Obsessive–compulsive Disorder
VDR: Vitamin D Receptor
RA: Retinoic acid
RXR: Retinoic Acid Receptor
DSM-5: Diagnostic and Statistical Manual of Mental Disorders
BMI: Body Mass Index
6-OHDA: 6-Hydroxydopamine
